# Frequency patterns of core constipation symptoms among the Asian adults: a systematic review

**DOI:** 10.1186/s12876-017-0672-z

**Published:** 2017-11-02

**Authors:** Abdul Wahab Patimah, Yeong Yeh Lee, Mohd Yusoff Dariah

**Affiliations:** 1 School of Health Sciences, Universiti Sains Malaysia, Kubang Kerian, Kota Bharu, 16150 Kelantan Malaysia; 2 School of Medical Sciences, Universiti Sains Malaysia, Kubang Kerian, Kota Bharu, 16150 Kelantan Malaysia

**Keywords:** Adult, Asia, Constipation, Irritable bowel syndrome, perception

## Abstract

**Background:**

In clinical practice, assessment of constipation depends on reliability, consistency and frequency of several commonly reported or core symptoms. It is not known if frequency patterns of constipation symptoms in adults are different between the West and the East. This review aimed to describe core constipation symptoms and their frequency patterns among the Asian adults.

**Methods:**

Articles published in PubMed, MEDLINE, CINAHL and Science Direct from 2005 to 2015 were searched systematically. Studies were included if constipation satisfied the Rome II and or III criteria. Study populations consisted of Asian adults above 18 years old and with sample size above 50.

**Results:**

Of 2812 articles screened, 11 met the eligibility criteria. Constipation among Asian adults was characterized by three core symptoms of ‘straining’ at 82.8%, ‘lumpy and hard stool’ at 74.2% and ‘sensation of incomplete evacuation’ at 68.1% and the least frequent symptom was ‘manual maneuver to facilitate defecation’ at 23.3%. There was heterogeneity in frequency patterns of core symptoms between different Asian studies but also differences in core symptoms between constipation subtypes of functional constipation and irritable bowel syndrome with constipation.

**Conclusions:**

In general, Asian adults perceive constipation symptoms in a similar but not equivalent manner to the West. Recognition of core symptoms will increase the diagnostic confidence of constipation and its subtypes but more studies of the various specific Asian populations are needed to address their differences.

## Background

Constipation is essentially a symptom-based gastrointestinal disorder. Very often, the term describes experience of ‘poorly’ moving bowels [1]. In adults, common reported symptoms include difficult defecation or infrequent stool passage, hard stools, straining, sensation of blockage, unproductive attempts to defecate or a feeling of incomplete evacuation [1, 2]. Constipation may be secondary to an underlying disorder such as diabetes mellitus, hypothyroidism or cerebrovascular disease, or constipating drugs such as anticholinergic agents and analgesics. But more frequently there are no apparent disorders e.g. functional constipation (FC) and it may affect all ages with a clear prevalence in elderly [3]. The community prevalence of self-reported constipation in Asia (especially in South East Asia countries) is lower compared to other parts of the world (range 1.4–32.9% in Asia vs. 0.7–79% for the rest of the world) [4, 5]. With Rome criteria, the prevalence of constipation is generally lower, for example, in Malaysia, the prevalence of irritable bowel syndrome (IBS) based on the Rome III criteria was 10.9% and out of this, 20% was IBS with predominant constipation (IBS-C) [6].

A patient’s experience with constipation is dynamic because it relates to interactions within their environment that aligned with their centered-care principles [7]. Weiss and Tyink explained this as a patient-centric culture, whereby people, place, personality and culture of the practice need to be in alignment with the ideal patient experiences [8]. As each person may have different life experiences, thus, their reactions or perceptions towards certain symptoms may also differ. The nature of constipation, severity, and duration of symptoms, as well as individual’s personal belief system are among the factors that may influence one’s perception towards constipation [9]. If constipation is treated as a subjective symptom then diagnosis, treatment and evaluation should be guided by patients’ perception and experience [10]. Sufferers of constipation believe the symptoms can affect their daily life significantly [11]. Beside constipation, the importance of symptom perception is also stressed in other clinical situations. For example, in atrial fibrillation, patient perception of their prevailing rhythm is often inaccurate and this reduces effectiveness of symptom-targeted treatment [12].

In general, self-reported symptoms, assistive measures including digital evacuation of stool and the use of laxatives are helpful to determine presence of constipation [13]. Self-reported constipation depends on the reliability and consistency of symptom perception [13]. Examples of common self-reported measures include Constipation Assessment Scale [14] and the Chinese Constipation Questionnaire [15]. Till date, there is a lack of reported studies on perception of core constipation symptoms among the Asians, unlike the West [16, 17]. This review aimed to describe core constipation symptoms and their frequency patterns in Asian adults.

## Methods

The Cochrane Collaboration’s recommendation for systematic review [18] and the Preferred Reporting Items for Systematic Reviews and Meta-Analyses (PRISMA) items were used where relevant as the basis to review the articles [19].

### Literature search

A comprehensive computerized database search was conducted from four electronic databases including PubMed, CINAHL, MEDLINE, and Science Direct from 2005 until 2015. The MeSH terms used included a combination of the following terms; ‘constipation’, ‘functional constipation’, ‘symptoms’, ‘irritable bowel syndrome’, ‘gastrointestinal diseases’, ‘defecation disease’ AND ‘Asia’. Bibliographies of retrieved articles were searched for additional studies.

### Study selection and analysis

**Fig. 1:**
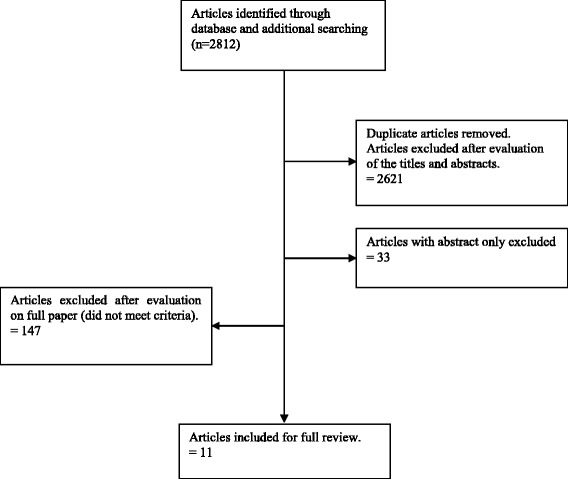
PRISMA flow diagram showing process of selection articles to include in review

Articles included in this review were considered appropriate for review if the following criteria were fulfilled: (i) constipation symptoms were based on the Rome II and III criteria. Briefly, with the Rome III criteria, those with constipation had symptoms for the last 3 months with onset of at least 6 months prior to the diagnosis but with Rome II criteria, the symptoms were present for at least 3 months, in the preceding 12 months. The symptoms are straining, lumpy or hard stool, sensation of incomplete evacuation, sensation of anorectal blockage, manual maneuver to facilitate defecation and having less than three defecations per week [20] (ii) the study population were Asian adults and at least 90% of the population aged 18 and above and with study sample size >50 (iii) full articles written in English. All study types were eligible for this review except case reports. Unpublished articles and studies in a language other than English were excluded. Of 2812 articles screened, 2654 were excluded upon scrutiny of titles and abstracts by the investigator (PAW). All authors read all 158 studies and assessed in more details. Finally, 11 eligible articles were included in this review (Fig. 1).

There were two possible diagnoses based on reported symptoms of constipation i.e. functional constipation (FC) and IBS-C [21]. Abdominal pain or discomfort was the primary symptom that distinguishes IBS from FC [22].

### Data extraction

For interventional or randomized studies with a pre- and post-evaluation, for example clinical trial, the pre-evaluation data were extracted. A core symptom was defined as the most prevalent symptom reported by participants. The symptoms were also ranked (first, second, third and so on) based on their frequency. Risk of bias (selection and reporting biases) in non-randomized studies were assessed using appropriate tools where available [23]. These biases might be a result of sampling population, study design and diagnostic method of constipation among others. For clinical trials, the risk of bias included selection bias, performance bias, detection bias, attrition bias, and reporting bias among others.

The Newcastle-Ottawa Scale (NOS) was modified to assess the quality of cross-sectional studies and other non-randomized studies included in our review [24]. Briefly, the quality was determined by the number of stars given for each of the three assessed categories i.e. selection (maximum three stars), confounders (maximum two stars) and outcome (maximum one star) (Table 1). In this review, the most important confounders were secondary chronic constipation/organic and metabolic disease and additional confounders were sex, elderly and lifestyles. The Cochrane Collaboration’s risk of bias assessment tool was used to assess the quality of clinical trials included in this review [18] (Table 2).

**Table 1:** Quality assessment of cross-sectional studies included in review

Study	Selection^a^	Confounders^b^	Outcome^c^	Total star	Quality^d^
Xin et al. (2014)	* *	*	–	3/6	Satisfactory
Zhao et al. (2011)	* * *	*	–	4/6	Good
Dong et al. (2013)	* *	*	–	3/6	Satisfactory
Yao et al. (2012)	* *	*	–	3/6	Satisfactory
Lu et al. (2006)	* *	* *	–	4/6	Good
Lee et al. (2014)	* *	*	–	3/6	Satisfactory
Gonlachanvit & Patcharatrakul (2005)	* *	*	–	3/6	Satisfactory
Kaboli et al. (2010)	* * *	*	*	5/6	Very good
Shalmani et al. (2011)	* * *	*	*	5/6	Very good
Roshandel et al. (2006)	* *	*	*	4/6	Good

^a^Selection category included assessment of representativeness of sample, non-respondents and ascertainment of constipation

^b^Confounders category included assessment of most important confounding factor and any additional factor

^c^Outcome category included assessment of outcome whether blinded, record-linkage, self-report or no/not clear description

^d^Quality of studies based on total stars given for all three assessed categories:

5 to 6 *: Very good studies

4 *: Good studies

3 *: Satisfactory studies

0 to 2 *: Unsatisfactory studies

**Table 2:** Quality assessment of randomized controlled trial included in review

Randomized controlled trial study	Jayasimhan et al	Support for the authors judgment
1. Selective	+	Quote: “Subjects were randomized using the sealed envelope method to either the treatment or placebo group…The placebo sample was similar in appearance and composition…”
a) Random sequence generation		
b) Allocation concealment	+	
2) Performance	+	Quote: “Patients and researchers were blinded to the allocated groups and the treatment allocation was revealed at the end of the research, once analysis was done” Patient returned home and would be reviewed for the outcome in the next appoinment (after 7 days)
a) Blinding of participants and personnel		
3) Detection	+	Unlikely the blinding could have been broken. Quote: “Follow-up was done at the end of the study period based on a questionnaire which includes symptomatic improvement and a stool diary”
a) Blinding outcome assessment		
4) Attrition	+	Reasons for missing outcome data unlikely to be related to the true outcome. Quote: “A total of 120 subjects were recruited but 12 did not complete the study and were considered dropouts. Dropouts were due to loss to follow-up, consent withdrawal and non-compliance such as consuming <80% of the test samples, intake of antibiotics, laxatives or other probiotics during the treatment period”
a) Incomplete outcome data		
5) Reporting bias	+	The study protocol is available and all of the study’s pre-specified primary and secondary outcomes that are of interest in the review have been reported in the pre-specified way. Quote: “The protocol was approved by the Institutional Review Board (IRB) of the University Malaya Medical Centre (Reference no: 866.59)…CONSORT diagram of patient recruitment and analysis”
a) Selective reporting		
6) Other bias	?	No description of what was defined by ‘normal diet’ which is an important risk of bias especially when this study consists of more than one ethnic and elderly population.
Justification for risk of bias	+	Low risk of bias for most key domains

+ Low risk of bias, − High risk of bias,? Unclear risk of bias

## Results

### An overview of selected articles

All 11 selected articles were from the period between 2005 and 2015 (Table 3). Four studies were from China [25–28], three from Iran [29–31], and one study each from Taiwan [22], South Korea [32], Thailand [33], and Malaysia [34]. Four studies adopted the Rome II criteria [22, 27, 31, 33] and six studies adopted the Rome III criteria [26, 28–30, 32, 34]. One study adopted both Rome II and III criteria [25]. Three studies provided data from participants with IBS-C [22, 27, 28] and the rest from participants with FC. Two studies from Taiwan and China compared those participants with FC and IBS-C [22, 27] and a study from Iran [31] compared constipation symptoms with other symptoms of functional bowel disorder.

**Table 3:** Selected Asian studies of core constipation symptoms

Reference/ Country	Sample /Age	Settings	Study design	Sampling	Sample size (response rate)	Diagnostic criteria
Xin et al. (2014), China	Chronic constipation patient, 18–80 years old	One clinic	Cross-sectional	Purposive	184 (100%): 166FC (Rome II) 174 FC (Rome III)	Rome II & Rome III
Zhao et al. (2011), China	Adult, 18–80 years old	5 regions of China	Cross-sectional	Randomized, stratified multistage	16,078 (89%): 948FC 183 IBS-C	Rome II
Dong et al. (2013), China	Volunteer students, 19–23 years old	One university	Cross-sectional	Random (study areas)	4638 (92.76%): 253 FC	Rome III
Yao et al. (2012), China	IBS patient, >20 years old	Three hospitals	Cross-sectional	Purposive	754 (97.2%): 108 IBS-C	Rome III
Lu et al. (2006),Taiwan	Volunteer adult, ≥20 years old	One hospital	Cross-sectional	Convenience	2018 (70.4%): 172 FC 54 IBS-C	Rome II
Lee et al. (2014), South Korea	Self-reported constipation adult, 20–89 years old	One National Health Screening Program	Cross-sectional	Random	625 (74.9%)	Rome III
Gonlachanvit & Patcharatrakul (2005), Thailand	Chronic constipation patients, 30 to 70 years old	One hospital	Cross-sectional	Purposive	103FC (100%)	Rome II
Jayasimhan et al. (2013), Malaysia	Outpatient, 20–78 years old	One clinic	Randomized-controlled trial	Purposive	108FC (90%)	Rome III
Kaboli et al. (2010), Iran	Households, ≥16 years old	5 suburb cities in one province	Cross-sectional	Random (postal code)	18,180 (92%): 459 FC	Rome III
Shalmani et al. (2011), Iran	Households, ≥16 years old	5 urban areas in one province	Cross-sectional	Cluster (postal code)	18,180 (94%): 435 FC	Rome III
Roshandel et al. (2006), Iran	Outpatient, >20 years old	One clinic	Cross-sectional	Purposive	1023 (100%): 115 FC 32 IBS-C	Rome II

### Quality of selected studies

In this review, heterogeneity among the studies was found mainly from the clinical and methodology aspects. Majority of studies were cross-sectional in nature and only one study was RCT. The RCT was included for review because it provided a frequency of symptoms of chronic constipation based on Rome III to evaluate effectiveness of their intervention. The total number of respondents included in this review was 3935, with 2933 FC, 377 IBS-C and 625 without specification of its subtypes. The samples were different between studies, consisting of patients with IBS and or FC, outpatients, general population and also students. Six studies had used non-randomized purposive or convenient sampling to recruit participants.

The quality of cross-sectional studies was between ‘very good’ and ‘satisfactory’ (mean stars 3.7, range 3–5) (Table 1), while for RCT, the bias was of low risk (Table 2). Specifically for cross-sectional studies, seven studies recruited their samples from selected users which might be a potential sampling bias. Four studies did not control confounders of pre-existing illnesses or secondary chronic constipation. While the other five studies did not include additional confounders or stated the method to reduce bias (e.g. statistical analysis). For the outcome category, most assessments were self-reported or providing unclear description.

### Symptoms of constipation

In general, symptoms of constipation were perceived between 10% and 98.4% of adult Asians (Table 4). The three core symptoms were ‘straining’ at 82.8%, ‘lumpy and hard stool’ at 74.2% and ‘incomplete evacuation’ at 68.1%. The least frequent symptom was ‘manual maneuver to facilitate defecation’ at 23.3%. The symptoms of ‘sensation of anorectal blockage’ and ‘infrequent defecation’ were intermediate in frequency at 47.4% and 59.1% respectively.

**Table 4:** Ranking of symptoms that are most commonly experienced in the Asian studies

Symptom	Rank 1st	Rank 2nd	Rank 3rd	Rank 4th	Rank 5th	Rank 6th
Straining	91.6% [25] 92.0% [25] 64.6% [32] 75.0% [27] 90.0% [29] 89.9% [30] 93.0% [33] *70.4%* [22] *65.0%* [27] *88.0%* [28]	95.7% [31] 79.3% [34] 70.3% [22]	93.7% [26]			
Sensation incomplete evacuation	72.1% [22] 96.5% [31]	64.2% [32] 94.9% [26] 69.0% [28] 87.0% [33] *64.8%* [22]	74.1% [34]	69.9% [25] 69.0% [25] 61.7% [29] 61.8% [30]	31.0% [27] *38.0%* [27]	
Lumpy/hard stool	93.1% [34]	70.0% [27] 85.8% [29] 86.2% [30] *65.0%* [27]	71.1% [25] 71.3% [25] 77.0% [33] 95.7% [31] *63.0%* [22]	58.9% [32] 38.4% [22] 88.5% [26]		
Sensation of anorectal blockage			53.5% [22]	46.0% [27] 62.6% [31] *46.3%* [22] *43.0%* [27]	53.0% [25] 52.3% [25] 39.5% [32] 87.7% [26] 26.6% [29] 26.2% [30] 31.0% [34] 48.0% [33]	
Infrequent defecations	98.4% [26]	74.7% [25] 74.7% [25]	58.9% [32] 56.0% [27] 66.0% [29] 66.0% [30] *57.0%* [27]	32.8% [34] 57.0% [33]	26.2% [22] *42.6%* [22]	57.4% [31]
Manual maneuver						18.1% [25] 18.4% [25] 14.8% [32] 47.0% [26] 10.0% [27] 26.1% [29] 16.3% [22] 16.1% [30] 53.9% [31] 15.5% [34] 45.0% [33] *11.1%* [22] *10.0%* [27]

*Italic,* symptom of IBS-C

The ranking of core symptoms, ‘straining’, ‘sensation of incomplete evacuation’, ‘lumpy and hard stool’ and ‘infrequent defecations’ were all rated as first in different studies but ‘sensation of anorectal blockage’ and ‘use of manual maneuver to evacuate stools’ were never ranked as first in all studies. ‘Straining’ was frequently rated as first, second or third rank, with the first rank (in eight studies) being the most common. Meanwhile, ‘lumpy and hard stool’ was usually ranked as first to the fourth, with the third rank (in four studies) being the most common and first rank (one study) the least. A similar picture was seen with the symptoms of ‘infrequent defecations’, with third rank (four studies) the most common. In contrast, the ‘sensation of incomplete evacuation’ was seen across almost all ranks except for the last with the second rank (five studies) being the most common and the third rank (one study) being the least.

Studies from Taiwan [22] and Iran [31] rated ‘sensation of incomplete evacuation’ as the most common constipation symptom at 72.1% and 96.5% respectively. However, in Malaysia, ‘lumpy and hard stool’ was more frequent at 93.1% rather than ‘straining’ at 79.3% [34]. Most studies from China and Iran also rated ‘infrequent defecation’ in the top three ranks with one study from China [26] rated it as the first at 98.4%. In contrary, studies from Malaysia and Thailand gave a lower rating for ‘infrequent defecation’ at 32.8% and 57% respectively. The symptom of ‘manual maneuver to facilitate defecation’ was consistently the least frequent in all Asian studies, ranging between 10% and 53.9%.

### Comparison between FC and IBS-C

Lu et al. from Taiwan showed that participants with IBS-C experienced more ‘infrequent defecation’ and ‘hard and lumpy stool’ than FC [22] (Table 5). On the other hand, Zhao et al. from China found that ‘incomplete evacuation’ was more commonly reported in IBS-C than FC and ‘straining’ was more common in FC than IBS-C [27]. Other symptoms were similar for both groups of participants. Despite the country differences, IBS-C and FC rated highly for ‘straining’ in contrast to ‘manual maneuver to facilitate defecation’ being the lowest.

**Table 5:** The difference of constipation symptoms between subjects with FC and IBS-C

Symptoms	Percentage (%)		*p*-value	Authors
	FC	IBS-C		
Straining	75	65	0.005	Zhao et al. (2011)
	70.3	70.4	N.S.	Lu et al. (2006)
	49	77.1	<0.001	Ford et al. (2014)
Sensation incomplete evacuation	31	38	0.045	Zhao et al. (2011)
	72.1	64.8	N.S.	Lu et al. (2006)
	44.3	70.9	<0.001	Ford et al. (2014)
Lumpy/hard stool	70	65	N.S.	Zhao et al. (2011)
	38.4	63	<0.001	Lu et al. (2006)
	45.5	81.7	<0.001	Ford et al. (2014)
Sensation of anorectal blockage	46	43	N.S.	Zhao et al. (2011)
	53.5	46.3	N.S.	Lu et al. (2006)
	31.2	56	<0.001	Ford et al. (2014)
Infrequent defecations	56	57	N.S.	Zhao et al. (2011)
	26.2	42.6	0.03	Lu et al. (2006)
	25.7	53.1	<0.001	Ford et al. (2014)
Manual maneuver	10	10	N.S.	Zhao et al. (2011)
	16.3	11.1	N.S.	Lu et al. (2006)
	14.3	32.6	<0.001	Ford et al. (2014)

*N.S.* not significant; a *p*-value < 0.05 is statistically significant

## Discussion

Six Asian countries namely China, Iran, Taiwan, South Korea, Thailand, and Malaysia are represented by the 11 studies included in this review. Diagnoses of FC and IBS-C were based on the Rome II and or Rome III criteria and both Rome II and III criteria have good agreement [25]. More Asian studies were available for FC than IBS-C. This review indicates that Asians perceived a range of symptoms at varying frequency from 10% to 98.4%. ‘Straining’ is perceived as the most frequent core symptom and ‘manual maneuver to facilitate defecation’ is the least reported core symptom in the Asian context and also regardless of whether the participants had FC or IBS-C. On the other hand, in Western studies, these core symptoms differ in frequency from our Asian data (Tables 3 and 4) and their first three most frequent core symptoms in the West appear more consistent than in the East. These core symptoms of Western studies are shown in Tables 6 and 7 and further comparisons between the two populations are discussed below.

**Table 6:** Selected Western studies of core constipation symptoms

Reference/ Country	Sample /Age	Settings	Study design	Sampling	Sample size (response rate)	Diagnostic criteria
Neri et al. (2016), Italy	Chronic constipation patient, mean age 50.1 (SD, 16.7)	39 Italian referral centers for gastrointestinal disorders	Cross-sectional	Purposive	2203	Rome III
Enck et al. (2016), German	Household adults, mean age 51.3 (SD, 0.6)	Telephone registry	Cross-sectional	RLD	589 (56.8%)	Rome III
Neri et al. (2014), Italy	Chronic constipation patient, mean age 50.3 (SD: 16.6)	39 Italian referral centers for gastrointestinal disorders	Cross-sectional	Purposive	856	Rome III
Ford et al. (2014), Canada	Referral patients, ≥16 years old	2 GI outpatient clinics of two hospitals	Cross-sectional	Purposive	3656 (86.6%) 343 FC 175 IBS-C	Rome III
Bellini et al. (2013), Italy	Chronic constipation patient, ≥18 years old	Primary care settings in Province Pisa represented by 41 GPs.	Cross-sectional	Stratified cluster	229 147 FC 50 IBS-C 32 SPC	Rome III
Johanson & Kralstein (2007), USA	Adults, ≥18 years old	Membership in the Knowledge Networks Panel	Cross-sectional	Purposive	557	Rome II
Pare et al. (2001), Canada	Household members, ≥18 years old	5 regions	Cross-sectional	Stratified random	1149 (57%)	Rome II

*FC* functional constipation, *GP* grand practitioners, *RLD* Random last digit, *GI* gastrointestinal, *SD* standard deviation

**Table 7:** Ranking of symptoms that are most commonly experienced in the Western studies

Symptom	Rank 1st	Rank 2nd	Rank 3rd	Rank 4th	Rank 5th	Rank 6th
Straining	82.3% [68]	*77.1%* [40]	41.9% [69]			
	79.0% [36]		*88.0%* [42]			
	82.2% [70]					
	81.0% [71]					
	49.0% [40]					
	81.6% [42]					
Sensation incomplete evacuation	74.2% [69]	68% [42]	72.8% [68]	54.0% [36]		
			73.8% [70]	*80.0%* [42]		
			54.2% [71]			
			44.3% [40]			
			*70.9%* [40]			
Lumpy/hard stool	*81.7%* [40]	74.4% [68]		61.9% [42]	33.2% [69]	
	*100.0%* [42]	71.0% [36]				
		74.8% [70]				
		71.5% [71]				
		45.5% [40]				
Sensation of anorectal blockage		53.9% [69]		38.8% [71]	40.4% [68]	*30.0%* [42]
				31.2% [40]	40.4% [70]	
				*56.0%* [40]	10.9% [42]	
Infrequent defecation		*100.0%* [42]	57.0% [36]	68.2% [68]	35.6% [71]	21.4% [69]
			68.0% [42]	64.3% [70]	25.7% [40]	
					*53.1%* [40]	
Manual maneuver				40.7% [69]	*36.0%* [42]	24.5% [68]
						24.6% [70]
						28.4% [71]
						10.2% [42]
						14.3% [40]
						*32.6%* [40]

*Italic,* symptom of IBS-C

In clinical practice, healthcare providers always emphasize the number of defecations in their constipated patients [35] with less attention paid to defecation symptoms. Knowledge of core individual symptoms may help improve diagnosis of constipation in a similar fashion to heartburn and regurgitation in gastroesophageal reflux disease. In our review, ‘straining’, ‘sensation of incomplete evacuation’ and ‘hard and lumpy stool’ are more consistent core symptoms in that order among Asians. While straining is similar, ‘hard and lumpy stool’ is more frequent than ‘sensation of incomplete evacuation’ in the West.

A study pointed out the three core symptoms of constipation in their population were ‘straining’, ‘gas’ and ‘hard stool’ [36]. In our review, ‘gas’ or bloating or distention was not reported because these symptoms are absent in the Rome criteria [21]. However, a study by Roshandel et al. found that 73% of their FC subjects also had symptom of fullness, bloating or visible distention [31]. Similar finding was reported by Gwee et al. from Singapore with bloating a feature in half of their constipated patients [37]. Several studies suggested that FC and IBS-C were not distinctive [38, 39] and bloating may indicate an overlap of both disorders. Ford et al. reported that bloating was the least frequent in those with FC but more frequent in those with IBS-C [40]. Further studies are needed to characterize bloating and distention in constipation.

Hard stools are among the most prevalent bowel complaints in the United States and United Kingdom and this is also shown in our review among Asians [41]. Besides difficulty in evacuation, hard and lumpy stools have been associated with delay in colonic transit [3]. Hard stools are frequently reported in those with IBS-C [40, 42] but age and ethnicity also affect its frequency. For example, Gwee and Setia from Singapore found that ‘hard and lumpy stool’ was more common among older adults over the age of 40 and above but ‘straining’ was usually reported by young people aged 18 to 29 years [43]. Similar to Singapore, ‘hard and lumpy stool’ is also a frequent core symptom in Malaysia [37] and this is because of their comparable ethnic backgrounds [44]. And because of similar ethnicity, constipation reported from China and South Korea is more consistent compared to Indonesia [5].

Cultural factors especially diet and also lifestyle factors are commonly associated with constipation in the Asian community [45]. A study from Bangladesh showed that low vegetable and spices intake were found to be associated with constipation [46]. In Japan, Singapore and Iran where rice is the staple food, studies showed that decreased intake of rice was associated with constipation [29, 47–49]. In addition, Wong and colleagues in Singapore also found that those who drank Chinese tea tended to get constipation [48]. On the other hand in Australia, only 35% of the elderly with constipation perceived food to cause their constipation [50]. Taking vegetables and fruits in large amounts of diet and using a squat toilet were the reasons for the low prevalence of FC in Iran [29].

Increasing age and women gender are common factors that may influence perception of constipation [4, 32, 51–53]. Elderly are associated with a higher prevalence of constipation because of their underlying co-morbid diseases but they also experience more side effects from medicines [48, 54]. A higher prevalence of constipation in women is possibly because of dynamic changes in their sex hormones and gynecological function [55]. Constipation is associated with hormonal changes that alter the gut function on the first day of menstruation [56, 57] while progesterone increases the colon transit time during pregnancy [58]. However, hormonal mechanism is not always clear cut as a study of 253 women before menopause and 252 men below age 50 asserted that bowel symptoms were more frequent in women than in men, regardless of menstrual phases [59].

A relatively high percentage of ‘infrequent defecation’ has been observed in our current review among Asians although this symptom was not highly rated in reported studies. ‘Infrequent defecation’ is associated with delay in colonic transit [33] and the delay usually occurs because of ageing [60]. Therefore, ‘infrequent defecation’ is not a core constipation symptom in patients with normal transit constipation and anorectal dysfunction [33]. However, a study by Roshandel et al. was inconsistent compared to other studies where almost all constipation symptoms (including infrequent defecation) were highly rated [31]. This might be due to their over-representation of female gender [31, 53, 60].

In the present review, it was not known if all subjects in the studies who met the Rome criteria for constipation were also aware or actually perceived they had constipation. A study from Hong Kong has shown that only 57% of patients were aware of their constipation [11]. This also suggests that a significant number of patients may not actually realize that they have constipation. However, those who self-reported constipation are more likely to have real constipation than those who fulfilled the Rome criteria alone [45, 48, 52]. Therefore, in addition to core symptoms, the diagnosis of constipation may be more reliable when the patients themselves also self-report constipation [61]. However, there is not always an agreement between subtypes of constipation. For example, a study showed that self-reported-constipation had a good correlation with Rome III criteria but there was no agreement with FC and IBS-C diagnoses [62]. A recent study in Italy also showed that less than 40% of patients were referred for chronic constipation fulfill either FC or IBS-C [42, 63].

It must be noted that while assistive measure of constipation for example digital evacuation of stool is mentioned as a diagnostic criterion in Rome III but this symptom is rarely reported and may be mis-interpreted. Johanson and Kralstein did not include ‘the need for manual maneuvers to facilitate defecation’ as a criterion for constipation in their study, because of a high degree of misinterpretation among patients which may mislead the result [36]. In our review, Asian adults also rarely perceived ‘manual maneuver to facilitate defecation’ as a core constipation symptom but whether this is because of social reason or that it is rarely performed needs further studies. Jayasimhan et al. stated that this symptom could indicate a more severe spectrum of chronic constipation [34]. Meanwhile, a frequent symptom experience suggested the presence of anorectal dysfunction [25, 64].

Some limitations need to be highlighted. Studies that were observational in nature could be prone to biases in sample selection, confounding factors and measurement tools. However, none of the selected studies were unsatisfactory based on the Newcastle-Ottawa scale. When using the Rome criteria to diagnose constipation, the studied population should have a similar understanding of the word used to describe constipation [65]. However, this is not always the case because of cross-cultural differences between countries and therefore the frequency of symptoms reported in Asian studies may be under- or over-reported. There are only a few Asian countries included in this review and China and Iran have larger sample sizes than other Asian populations. In addition, we suspect there may be some heterogeneity among different Asian populations and further studies in the future should probably be population-specific rather than generalized to the East or to the West. Even within a country, there may be differences, for example, a study conducted in different areas in China found that low socio-economic status and dry weather resulted in more reports of constipation [66]. Factors that can influence perception of constipation such as genetic, environment, psychosocial, physiology and clinical outcome [67] should be taken into account in addition to self-reported constipation.

## Conclusion

In conclusion, our review indicates that Asians perceive constipation in a similar but not in an equivalent manner to the West. Symptoms in the Rome criteria are also experienced by Asians but there is heterogeneity in frequency and patterns of core symptoms. Recognition of core symptoms will increase the diagnostic confidence of health care providers in their clinical practice. More studies of the various specific populations within Asia are needed to address their differences.

## Abbreviations

FC: Functional Constipation
IBS: Irritable bowel syndrome
IBS-C: Irritable bowel syndrome with predominant constipation
MeSH: Medical Subject Headings
NOS: Newcastle-Ottawa Scale
PRISMA: Preferred Reporting Items for Systematic Reviews and Meta-Analyses
RCT: Randomized clinical trial
